# Molecular Typing of *Strongyloides stercoralis* from an Iranian Patient

**Published:** 2019

**Authors:** Narges KALANTARI, Tahmineh GORGANI-FIROUZJAEE, Mostafa JAVANIAN, Salman GHAFFARI

**Affiliations:** 1. Cellular and Molecular Biology Research Center, Health Research Institute, Babol University of Medical Sciences, Babol, Iran; 2. Infectious Diseases and Tropical Medicine Research Center, Health Research Institute, Babol University of Medical Sciences, Babol, Iran; 3. Department of Mycology and Parasitology, School of Medicine, Babol University of Medical Sciences, Babol, Iran

**Dear Editor-in-Chief**

*Strongyloides stercoralis*, a soil-transmitted helminth, is one of the most important gastrointestinal parasites. Due to its complex life cycle, particularly endogenous autoinfection, this parasite is able to develop into its host, which leads to the persistence of chronic infection for several decades or results in hyperinfection. It is also found in other mammals including dogs, considered as a zoonotic infection (1).

However, recent studies using mitochondrial locus cytochrome c oxidase subunit 1 (cox1) gene and the whole genome sequence of individual *S. stercoralis* indicate substantial genetic diversity among *S. stercoralis* isolated from humans and dogs (1). Five, two and seventeen different haplotypes were reported based on HVR I and HVR IV of the SSU and Cox1 genes (2). Moreover, 100 haplotypes have been reported from 571 isolates using Cox1 sequences (3).

To the best of our knowledge, there is no documented data on *S. stercoralis* genotypes and the zoonotic transmission of this parasite to humans in Iran. The aim of the present study was the molecular typing of a *S. stercoralis* isolated from a patient with gastrointestinal manifestations from Babol, northern Iran.

Fresh stool samples was obtained to isolate the *S. stercoralis* rhabditiform larvae using the agar plate technique (4). Total DNA was extracted from the larvae using a commercial kit according to the manufacturer’s instructions (PCRBIO Rapid extract PCR kit, UK). A conventional PCR was performed to amplify a 650 bp fragment of the *S. stercoralis* Cox1 gene using specific primers as previously described (1). We used H_2_O as a negative control. The PCR product was sequenced in one direction (ABI 3730XL DNA Analyzer, Bioneer Company, South Korea). The sequences were analyzed with Chromas (v.2.6.4) software and compared with the reference sequence in GenBank database (https://www.ncbi.nlm.nih.gov/genbank/). A multiple sequence alignment was generated using the ClustalW alignment tool of the MEGA v.6. The maximum likelihood method and 1000 bootstrap replicates were used to perform phylogenetic analysis (5, 6).

A high level of similarity (99%) was observed between the partial sequence of the Cox1 genes obtained in our work and the key reference sequences deposited in GenBank from Myanmar (accession No. KX226368 (3)).

**Fig. 1:**
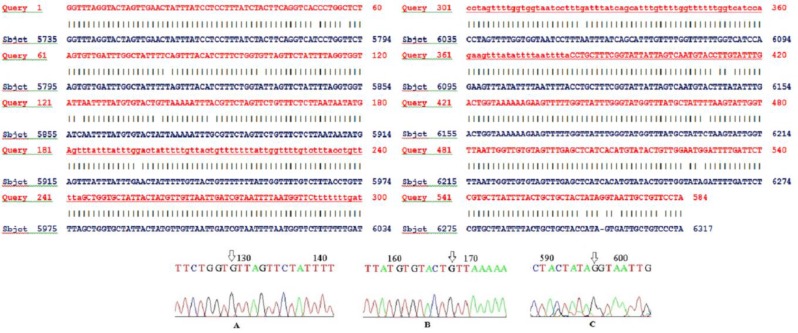
Partial chromatogram of the mitochondrial Cox1 (650 bp) sequence of the studied *S. stercoralis* isolate compared with reference sequence alignment (accession number:LC050212). Arrow shows a transversion mutations of adenine to guanine in positions 5834 and 5873 (A and B), and insertion of guanine in position 6302(C)

**Fig. 2:**
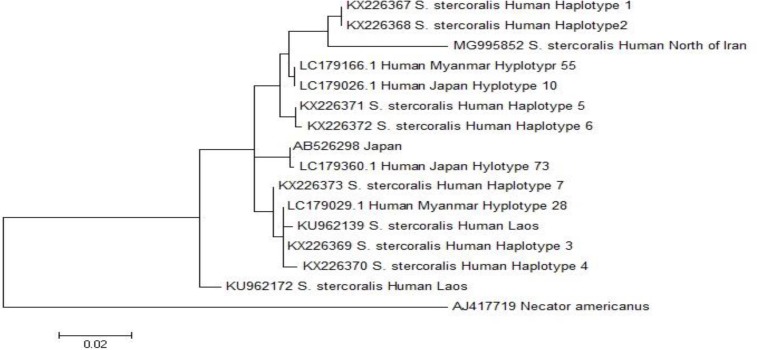
Maximum-likelihood tree built for *Strongyloides stercoralis* isolate of a 650 bp fragment of the Cox1 gene using MEGA6 software. Scale bar represents 0.02 changes per nucleotide site. *Necator americanus* was used as an out group in this study

Eighteen point mutations were observed in the partial sequence of the Cox1 gene compared to the reference sequence (LC050212.1). Substitution mutation of thymine (T) to cytosine (C) in positions 5785, 5791, 5819 and 6145, T to adenine (A) in position 6259, A to guanine (G) in positions 5834, 5873, 5929, 6058, 6148 and 6262, G to A in positions 5884 and 6305, C to T in position 5818, 6203, 6298 and 6314, were seen. Besides, a G was inserted at position 6302. Two out of 18 point mutations were non-synonymous, which occurred in positions 5834 and 5873 (A to G) causing valine to substitute isoleucine. The position of these point mutations and the insertion of the guanine are shown in Fig. 1. The multiple sequence alignments and phylogenetic analysis compared with different human haplotypes of *S. stercoralis* showed that this isolate is collapsed to human haplotype 1&2 (1, 3). This isolate is a new haplotype with seven point mutations in the partial sequence of the Cox1 gene (Fig. 2). This sequence was deposited in GenBank with the accession no MG995852 (7).

In conclusion, based on our best information, this is a new haplotype of *S. stercoralis* from Iran. Further genotype study of *S. stercoralis* isolates to better understand the phylogenetic relationships among *S. stercoralis* isolate, and also to evaluate the potential of zoonotic transmission is recommended.
